# Determinants of delayed diagnosis and treatment of tuberculosis in Cambodia: a mixed-methods study

**DOI:** 10.1186/s40249-020-00665-8

**Published:** 2020-05-07

**Authors:** Alvin Kuo Jing Teo, Chetra Ork, Sothearith Eng, Ngovlyly Sok, Sovannary Tuot, Li Yang Hsu, Siyan Yi

**Affiliations:** 1 Saw Swee Hock School of Public Health, National University of Singapore, National University Health System, Singapore, Singapore; 2 KHANA Center for Population Health Research, Phnom Penh, Cambodia; 3 Yong Loo Lin School of Medicine, National University of Singapore and National University Health System, Singapore, Singapore; 4 Center for Global Health Research, Touro University California, Vallejo, USA; 5 School of Public Health, National Institute of Public Health, Phnom Penh, Cambodia

**Keywords:** Tuberculosis, Delayed diagnosis, Health-seeking behavior, Cambodia

## Abstract

**Background:**

Cambodia is among the 30 countries in the world with the highest burden of tuberculosis (TB), and it is estimated that 40% of people with TB remain undiagnosed. In this study, we aimed to investigate the determinants of delayed diagnosis and treatment of TB in Cambodia.

**Methods:**

This mixed-method explanatory sequential study was conducted between February and September 2019 in 12 operational districts in Cambodia. It comprised of a retrospective cohort study of 721 people with TB, followed by a series of in-depth interviews. We assessed factors associated with time to TB diagnosis and treatment initiation using Cox proportional hazards model. Subsequently, we conducted in-depth interviews with 31 people with TB purposively selected based on the time taken to reach TB diagnosis, sex, and residence. Transcripts were coded, and thematic analyses were performed.

**Results:**

The median time from the onset of symptoms to TB diagnosis was 49 days (Interquartile range [IQR]: 21–112). We found that longer time to diagnosis was significantly associated with living in rural area (Adjusted hazards ratio [a*HR]* = 1.25; 95% confidence interval [*CI*]: 1.06–1.48); TB symptoms—cough (a*HR*: 1.52; 95% *CI*: 1.18–1.94), hemoptysis (a*HR* 1.32; 95% *CI*: 1.07–1.63), and night sweats (a*HR*: 1.24; 95% *CI*: 1.05–1.46); seeking private health care/self-medication (a*HR*: 1.23; 95% *CI*: 1.04–1.45); and higher self-stigma (a*HR*: 1.02; 95% *CI*: 1.01–1.03). Participants who received education level above the primary level were inversely associated with longer time to diagnosis (a*HR*: 0.78; 95% *CI*: 0.62–0.97). The median time from TB diagnosis to the initiation of treatment was two days (IQR: 1–3). The use of smear microscopy for TB diagnosis (a*HR*: 1.50; 95% *CI*: 1.16–1.95) was associated with longer time to treatment initiation. Seeking private health care and self-medication before TB diagnosis, lack of perceived risk, threat, susceptibility, and stigma derived qualitatively further explained the quantitative findings.

**Conclusions:**

TB diagnostic delay was substantial. Increasing public awareness about TB and consciousness regarding stigma, engaging the private healthcare providers, and tailoring approaches targeting the rural areas could further improve early detection of TB and narrowing the gap of missing cases in Cambodia.

## Background

Tuberculosis (TB) is a leading infectious cause of morbidity and mortality worldwide, accounting for 10 million new cases and 1.2 million deaths in 2018 [1]. Cambodia is one of the 30 countries with the world’s highest burden of TB, with an estimated incidence of active TB of 302 (95% *CI*: 169–473) per 100 000 population in 2018 [1]. Led by the National Center for Tuberculosis and Leprosy Control (CENAT), the national TB control program was set up to control and treat TB, including the introduction and country-wide expansion of directly observed therapy short-course (DOTS) since 1994 [2]. As in many other high TB-burden countries, passive case finding (PCF) is the default setup for TB case finding at health centers where people with TB symptoms seek care, and the providers can identify the conditions [3]. This strategy is, however, inadequate to detect and measure the burden of undiagnosed TB in the community. Active case finding (ACF), a strategy that has gained traction in recent years, was found to increase case detection in Cambodia [4]. Yet, despite increased efforts to find the missing cases, every fourth person with TB goes undetected in Cambodia [1], similar to the proportion reported globally in 2018 [1].

Prolonged delays have been associated with further transmission of the infection in the community [5] and thus posed a great challenge to TB elimination efforts globally [6, 7]. Therefore, understanding the specific determinants of delayed diagnosis can be used as a practical guide to enhance outreach programs, increase community engagement to reach missing cases, and to improve TB control strategies. Recent systematic reviews have reported empirical evidence associating socio-demographic, clinical, health system, and economic factors with delayed diagnosis and treatment of TB [8–10]. In Cambodia, Sundaram and colleagues have reported that strong preference for private healthcare services, lack of awareness of TB symptoms, and misbeliefs regarding TB (such as TB is a hereditary disease and the ability to recover without treatment) may delay seeking TB diagnosis [11]. However, the socio-cultural and clinical determinants of delayed diagnosis have yet to be thoroughly examined. Furthermore, other individual-level factors such as stigma have been associated with health inequalities and a barrier to health care [12], but its impact on health-seeking decisions and TB diagnosis were inconclusive [13, 14]. In this study, we aimed to explore the determinants of delayed diagnosis and treatment of TB in Cambodia.

## Methods

We applied a mixed-method explanatory sequential study design comprising a retrospective cohort study, followed by in-depth interviews (IDIs) with people with TB in Cambodia. The National Ethics Committee for Health Research Cambodia (NECHR) (NECHR reference: 024/NECHR) and National University of Singapore (NUS) Institutional Review Board (IRB) (NUS IRB reference: H-19-015) approved the study. Informed consent was obtained from all respondents before study enrolment.

### Retrospective cohort study

#### Setting

We conducted a retrospective cohort study on people with TB in 12 operational districts (OD) in 10 provinces in Cambodia. We selected 100 health centers with a probability proportional to size (by the total population each health center served) sampling without replacement from the total number of health centers (*n* = 143) in the 12 ODs.

#### Study population

In the selected ODs, trained data collectors recruited participants aged 18 and above who were diagnosed with TB within 1 month of survey implementation, regardless of the history of previous TB treatment, HIV status, and the drug resistance status. Bacteriological status of participants was determined by either smear microscopy or GeneXpert MTB/RIF. For participants who were tested negative, further assessment and TB diagnosis were made by clinicians based on clinical and laboratory grounds, and chest radiographic abnormalities [15]. TB workup and diagnoses were done in accordance with the national guideline [15]. We excluded those who refused to participate.

#### Key variables and definition

We collected information on socio-demographic characteristics, presence of other known medical conditions (one or more co-morbidities would constitute a yes versus no), TB symptoms before diagnosis (and the date of onset), knowledge and beliefs on TB, TB-related stigma experiences, and psychological distresses using a paper-based questionnaire. We asked if study participants had sought healthcare prior to TB diagnosis and the facilities that they had visited. In this study, private health care facilities included private pharmacies, private general practitioners, private hospitals, and traditional healers. Self-medication referred to the use of medications without professional advice. Public health facilities referred to government hospitals and health centers. We dichotomized the level of residence urbanization into “urban” and “rural” based on the Ministry of Planning’s framework [16, 17]. Using the WHO TB knowledge, attitude, and practices survey [18] and the General Health Questionnaire (GHQ)-12 [19, 20], we measured participants’ TB knowledge and the psychological distress they experienced before TB diagnosis, respectively (Supplementary Materials). Study participants who scored above the median [21] were regarded as having good TB knowledge.

We measured TB stigma using validated scales developed by Van Rie and colleagues [22, 23]. Self-stigma (12 items) and perceived stigma by the community (11 items) were measured on a Likert scale of 0 to 3 (0 = “strongly disagree” and 3 = “strongly agree”). The scales measured thoughts and feelings of people with TB (self-stigma) and the perceptions of people with TB regarding how the community feels about people with TB (perceived stigma by the community) [22]. Based on the four-point Likert scale, the minimum score was 0, and the maximum possible score was 36 and 33, respectively. Summary stigma scores were standardized to 50 with higher scores indicating a higher level of stigma.

The main outcomes of interest in this study were diagnostic and treatment delay. We defined diagnostic delay as the duration (time in days) between the onset of symptoms first recognized and self-reported by study participants and the final diagnosis of TB. Treatment delay was defined as the duration (time in days) between TB diagnosis and treatment initiation.

#### Data collection procedures and quality assurance

Trained data collectors conducted face-to-face interviews at the health centers with study participants using a paper-based questionnaire. We pre-tested the questionnaire with eight people with TB and TB survivors (data excluded from the main study). Data collectors were trained to use prompts such as significant cultural and public holidays in Cambodia to aid participants in recalling the most accurate date of onset of TB symptoms possible. Participants were reimbursed with United States dollar (USD) 5 at the end of the interview. We obtained information on the final diagnosis of TB and treatment initiation date from the facilities where TB diagnosis was made. To ensure the quality of the data, the study team conducted several supervisory field trips, and teleconference meetings with the field team were held fortnightly. Before data entry, the questionnaires were checked for completeness. Four members of the research team entered data in the questionnaires into KoBoToolbox (Harvard Humanitarian Initiative, Cambridge, Massachusetts, USA) [24]. Regular checks on the entered data were conducted. Inconsistencies were discussed, and discrepancies were resolved by revisiting the original questionnaires. Data were exported into Microsoft Excel (Microsoft Office Professional Plus 2016, Microsoft Corp., Redmond, Washington, USA) for data cleaning before analyses.

#### Statistical analyses

First, we described and presented the data using frequencies and percentages for categorical variables and median with interquartile range (IQR) or mean with standard deviation (*SD*) for continuous variables. We assessed the determinants of diagnostic and treatment delay using time-to-event analyses. The time to events —TB diagnosis and initiation of TB treatment—were measured in days. In this study, events were regarded to have occurred when a TB diagnosis was made, or TB treatment was initiated. As all the participants in this study were diagnosed with TB and initiated on treatment, no data were censored. However, participants (*n* = 100) who started on treatment on the same day (time to event = 0) as they were diagnosed were not included in the risk set. Log-rank test and univariate Cox proportional hazard regression were used to estimate the statistical significance of categorical and continuous variables, respectively. Epidemiologically relevant variables and other exposure variables with *P*-value ≤ 0.1 in univariate analyses were included in the Cox proportional hazard model. We checked model fit using Akaike Information Criteria [25] and assessed the assumption of proportional hazards using Schoenfeld global test of residuals [26], and no violations were observed (diagnostic delay model: *P* = 0.428, treatment delay model: *P* = 0.840). We evaluated the fit of the final models using Cox-Snell residuals. Hazard ratios (*HR*) were reported with 95% confidence interval (*CI*), and a 2-tailed *P*-value < 0.05 was considered significant. All data were analyzed using STATA 14 (StataCorp LP, Texas, United States of America).

### In-depth interviews

#### Study population

Study participants for the qualitative IDIs were purposively selected using maximum variation sampling from the cohort recruited in the survey described above. To ensure equal representation of perspectives from persons who experienced diagnostic delay and those who were diagnosed early in the course of their disease, we selected participants from the both extremes of the time to diagnosis spectrum— < 15 days (short delay) and > 100 days (long delay)—and stratified them according to sex (men/women) and residence (urban/rural). We approached 37 individuals, and six people refused to participate due to illness and work priorities. In total, 31 people with TB were interviewed.

#### Data collection

We developed a semi-structured interview guide that comprised of elements related to participants’ illness experiences, diagnosis, and treatment-seeking behavior. Questions and probes were focused on 1) knowledge and perception of TB as a disease; 2) experiences with seeking diagnosis and treatment for TB, with clinical and/or traditional approaches; and 3) barriers and facilitators experienced in seeking diagnosis and treatment. The semi-structured interview guide was pilot tested and revised accordingly. The IDIs were conducted in four ODs in September 2019 by CO, SE, and NS, who were trained in qualitative research. We matched the interviewers and interviewees by sex to minimize potential biases in participants’ responses. Most of the interviews were conducted 1∶1 in a private room at the health centers nearest to participants’ homes. We also conducted some interviews at participants’ homes, and on some occasions, family members were present. All the interviews were conducted in Khmer, and each interview lasted between 30 to 60 min. Interviews were audio-recorded, and field notes were taken during the interviews. Participants’ received USD 10 in return for their time and effort.

#### Data analyses

CO, SE, and NS transcribed all the IDIs verbatim. Transcripts in Khmer were then translated to English for analyses. The translated English transcripts were reviewed against the original version for verification by SE. Annotation and analysis of complete transcripts were conducted using NVivo (Version 10, QSR International, Burlington, Massachusetts, USA). Textual references to the topics of interest were retrieved and categorized using thematic analysis. AKJT and SE independently read and coded the transcripts characterized by 1) those with shorter delay to diagnosis and 2) those with a longer delay to diagnosis. We derived initial themes based on the semi-structured interview guide in developing a codebook of structural codes. We added emergent codes accordingly to the codebook. No new concepts relevant to the objective of this study were identified after interviewing 31 individuals, and data saturation were reached. Conclusions were drawn through the interpretation of the derived themes and the triangulation of qualitative data with quantitative findings.

## Results

### Retrospective cohort study

In total, 721 people with TB participated in this study (Table 1). The median age was 61 years (IQR: 52–71), and 36.9% were living in an urban setting. The cohort comprised of men (53.1%), individuals who were married (77.8%), and individuals who had at least primary education (84.5%). Forty percent of the participants were diagnosed with bacteriologically confirmed TB, and GeneXpert MTB/RIF test informed most of the TB diagnosis. The median distance from the place of residence to the nearest public health facility was four kilometers (IQR: 2–6). Sixty-six percent of the study participants sought private health care and/or self-medicated before TB diagnosis. The median knowledge score reported by study participants was 9. The mean scores for self-stigma and perceived stigma by the community were 25.1 (*SD* = 6.9) and 26.0 (*SD* = 7.2), respectively, and they were normally distributed. The Cronbach’s alphas for the self-stigma was 0.878 and for the perceived stigma by the community scale was 0.877.

**Table 1:** Participants characteristics and time from onset of symptoms to TB diagnosis

Variables	Median	IQR
Distance from home to health facility, in kilometer	4	2–6
Age, in years	61	52–71
	**Frequency**	**%**
Total participants	721	100.0
Residence		
Urban	266	36.9
Rural	455	63.1
Sex		
Male	383	53.1
Female	338	46.9
Education level^a^		
Primary and lower	605	84.5
Above primary	111	15.5
Marital status		
Never married	31	4.3
Currently married	561	77.8
Widowed/divorced	129	17.9
Ever smoked		
Ever (current and past smokers)	216	30.0
Never	505	70.0
Current smoker		
Current smoker	132	18.4
Not current smoker	587	81.6
Current alcohol use^a,b^		
Non-drinkers	509	71.1
Drinkers	207	28.9
Presence of other known medical conditions		
Yes	523	72.5
No	198	27.5
Type of TB		
Bacteriologically confirmed TB	284	39.4
Clinician diagnosed TB	437	60.6
Type of diagnostic tests performed		
GeneXpert	605	83.9
Smear microscopy	116	16.1
Cough		
Yes	638	88.5
No	83	11.5
Hemoptysis		
Yes	124	17.2
No	597	82.8
Fever		
Yes	391	54.2
No	330	45.8
Chills		
Yes	126	17.5
No	595	82.5
Weight loss		
Yes	482	66.9
No	239	33.1
Night sweats		
Yes	352	48.8
No	369	51.2
Health care seeking prior to TB diagnosis^c^		
Public health facilities	244	33.8
Private health facilities/self-medication	477	66.2
TB knowledge^d^		
Poor	179	24.8
Good	542	75.2
Perception about the seriousness of TB as a disease^a^		
Very serious	275	38.3
Not very serious	444	61.7
Self-perceived risk of getting TB^a^		
Yes, at-risk	382	56.8
No, not at-risk	291	43.2
Total General Health Questionnaire-12 score^e^		
≤ 3	364	50.5
> 3	357	49.5

*SD* Standard deviation, *IQR* Interquartile range, *TB* Tuberculosis

^a^ Exclude missing values

^b^ Drinkers reported frequency of alcohol use that ranges from once a month or less to 4 times or more per week. Non-drinkers refer to teetotalers

^c^ Participants were asked if they have sought health care prior to TB diagnosis and if so, the facilities that they have visited. Private health facilities referred to pharmacy, private general practitioner, and traditional healer. Self-medication referred to the use of medications without professional advice. Public health facilities referred to government hospitals and health centers

^d^ Evaluated based on the answers from 8 questions regarding the characteristics, symptoms of TB, route of transmission, prevention, and treatment of TB with a total score of 13 (median = 9). Respondents were regarded as having poor TB knowledge if they scored the median and below and good TB knowledge if they scored above the median

^e^ Evaluated based on the total score of the 6 negative items. Scoring method: 0–0–1–1, with 0 = “less than usual”, 0 = “no more than usual”, 1 = “rather more than usual”, or 1 = “much more than usual”

The median time from onset of symptoms to TB diagnosis was 49 days (IQR: 21–112). In univariate analysis, a longer time to TB diagnosis was significantly associated with TB symptoms (cough, hemoptysis, fever, weight loss, and night sweats), education level, seeking private health care/self-medication, poor TB knowledge, experiencing psychological distress after falling sick (higher GHQ-12 scores), higher self-stigma, and TB diagnosis informed by smear microscopy (Table 2). In the multivariate model adjusted for age, gender, and residence, a longer time to TB diagnosis remained significantly associated with rural residence (Adjusted hazards ratio [a*HR*] = 1.25; 95% *CI*: 1.06–1.48, *P* = 0.01); TB symptoms—cough (a*HR* = 1.52; 95% *CI*: 1.18–1.94, *P* = 0.001), hemoptysis (a*HR* = 1.32; 95% *CI*: 1.07–1.63, *P* = 0.01), and night sweats (a*HR* = 1.24; 95% *CI*: 1.05–1.46, *P* = 0.01); seeking private health care/self-medication (a*HR* = 1.23; 95% *CI*: 1.04–1.45, *P* = 0.01); and higher self-stigma (a*HR* = 1.02; 95% *CI*: 1.01–1.03, *P* = 0.003). Participants who received education level above the primary level were inversely associated with longer time to diagnosis (a*HR* = 0.78; 95% *CI*: 0.62–0.97, *P* = 0.03).

**Table 2:** Cox proportional hazard models by time (days) from onset of symptoms to TB diagnosis and from TB diagnosis to treatment initiation among people with TB in the study

Characteristics	Time from onset of symptoms to TB diagnosis (days)						Time from TB diagnosis to treatment initiation (days)					
	Crude *HR*	95% ***CI***	***P***-value	Adjusted ***HR***	95% ***CI***	***P***-value	Crude ***HR***	95% ***CI***	***P***-value	Adjusted ***HR***	95% ***CI***	***P***-value
Residence												
Urban	1.00			1.00			1.00			1.00		
Rural	1.46	1.25–1.70	< 0.001	1.25	1.06–1.48	0.010	0.92	0.78–1.08	0.296	0.95	0.80–1.13	0.562
Sex												
Male	1.00			1.00			1.00			1.00		
Female	1.02	0.88–1.18	0.760	0.91	0.77–1.06	0.217	1.05	0.90–1.23	0.517	1.06	0.90–1.24	0.504
Age, in years	1.00	1.00–1.01	0.657	1.00	0.99–1.00	0.259	1.00	0.99–1.00	0.642	1.00	0.99–1.01	0.604
Distance from home to the nearest public health facility, in kilometers	0.98	0.96–1.00	0.041	0.98	0.96–1.00	0.115	1.01	0.99–1.03	0.427			
Education level												
Primary and lower	1.00			1.00			1.00					
Above primary	0.70	0.57–0.86	0.001	0.78	0.62–0.97	0.026	1.03	0.82–1.30	0.775			
Cough												
Yes	1.00			1.00			1.00			1.00		
No	1.56	1.24–1.97	< 0.001	1.52	1.18–1.94	0.001	0.74	0.58–0.94	0.015	0.80	0.62–1.03	0.078
Hemoptysis												
Yes	1.00			1.00			1.00					
No	1.25	1.02–1.51	0.026	1.32	1.07–1.63	0.010	0.92	0.74–1.15	0.455			
Fever												
Yes	1.00			1.00			1.00					
No	1.28	1.10–1.48	0.001	1.14	0.97–1.34	0.113	0.88	0.75–1.04	0.125			
Weight loss												
Yes	1.00			1.00			1.00			1.00		
No	1.21	1.03–1.42	0.018	0.97	0.81–1.16	0.719	0.83	0.70–0.98	0.027	0.86	0.73–1.02	0.085
Night sweats												
Yes	1.00			1.00			1.00					
No	1.42	1.22–1.65	< 0.001	1.24	1.05–1.46	0.010	0.89	0.76–1.05	0.164			
Health care seeking prior to TB diagnosis^a^												
Public health facilities	1.00			1.00			1.00					
Private health facilities/self-medication	1.27	1.09–1.48	0.003	1.23	1.04–1.45	0.014	0.91	0.77–1.07	0.265			
TB knowledge^b^												
Poor	1.00			1.00			1.00					
Good	0.82	0.69–0.97	0.019	0.84	0.70–1.01	0.058	0.89	0.74–1.06	0.199			
Total General Health Questionnaire-12 score^c^												
≤ 3	1.00			1.00			1.00			1.00		
> 3	1.26	1.08–1.46	0.002	1.14	0.97–1.13	0.113	0.87	0.74–1.02	0.089	0.88	0.75–1.04	0.125
Self-stigma^d^	1.02	1.01–1.04	< 0.001	1.02	1.01–1.03	0.003	1.00	0.99–1.01	0.512			
Type of diagnostic test												
GeneXpert	1.00			1.00			1.00			1.00		
Smear microscopy	0.82	0.67–1.00	0.048	0.91	0.73–1.14	0.419	1.53	1.19–1.97	0.001	1.50	1.16–1.95	0.002

*HR* Hazards ratio, *CI* confidence interval, *ACF* Active case finding, *PCF* Passive case finding, *TB* Tuberculosis

^a^ Participants were asked if they have sought health care prior to TB diagnosis and the facilities that they have visited. Private health facilities referred to pharmacy, private general practitioner, and traditional healer. Self-medication referred to the use of medications without professional advice. Public health facilities referred to government hospitals and health centers

^b^ Evaluated based on the answers from 8 questions regarding the characteristics, symptoms of TB, route of transmission, prevention, and treatment of TB with a total score of 13 (median = 9). Respondents were regarded as having poor TB knowledge if they scored the median and below and good TB knowledge if they scored above the median

^c^ Evaluated based on the total score of the 6 negative items. Scoring method: 0–0–1–1, with 0 = “less than usual”, 0 = “no more than usual”, 1 = “rather more than usual”, or 1 = “much more than usual”

^d^ Evaluated based on the answers from 12 questions, measured on a Likert scale (0 to 3), with 0 being strongly disagree and 3 being strongly agree. Minimum score is 0 and the maximum possible score is 36. Summary stigma scores from were standardized to 50 with higher scores representing higher level of self-stigma

The median time from TB diagnosis to the initiation of treatment was two days (IQR 1–3). In univariate analysis (Table 2), a longer time to TB treatment initiation was significantly associated with TB symptoms (cough, and weight loss) and TB diagnosis informed by smear microscopy. In the multivariate model adjusted for age, gender, and residence, a longer time to treatment initiation remained significantly associated only with the use of smear microscopy for TB diagnosis (a*HR* = 1.50; 95% *CI*: 1.16–1.95, *P* = 0.002).

### In-depth interviews

Among the 31 IDIs, half were classified as having short delays and were living in an urban setting. The median age was 56 years (IQR: 45–68). We interviewed 18 men and 13 women (Table 3). An overview of the results is presented in Table 4.

**Table 3:** Characteristics of in-depth interviews participants

	Frequency	%
Age, in years (median, IQR)	56 (45–68)	
Sex		
Male	18	58.1
Female	13	41.9
Residence		
Urban	16	51.6
Rural	15	48.4
Time to TB diagnosis		
Short delay^a^	16	51.6
Long delay^b^	15	48.4

*IQR* Interquartile range, *TB* Tuberculosis

^a^ Time from onset of TB symptoms to TB diagnosis: < 15 days

^b^ Time from onset of TB symptoms to TB diagnosis: > 100 days

**Table 4:** Comparison of themes emerged from qualitative interviews between people with shorter delays and people with longer delays to TB diagnosis

	Themes		
	Similarities	Short delay	Long delay
Barriers to TB care-seeking	Lack of perceived risk and susceptibility to TB	Lack of perceived threat of TB to their own health	Sought private health care/self-medicated prior to TB diagnosis
	Unable to travel to health facilities due to intolerable symptoms or logistical constraints	Competing priorities – work, livelihood, and family responsibilities	
Facilitators to TB care-seeking	Encouragement from family members, and other TB survivors	Fear of infecting others	Intolerable symptoms
	Easy access to TB diagnosis provided by non-governmental organizations	Perceived illness would affect health, wellbeing, work, and livelihood	
Reasons for seeking private healthcare/self-medicate	Better access (facilities are closer to home)	Perceived illness to be less serious	Perceived to provide better care and service, and the medicines are more effective
Reasons for seeking public healthcare	Financially affordable	Perceived to provide better and safer care	Experienced good service in the past
TB stigma		Felt ashamed/embarrassed because of TB	Perceived stigma and discrimination against people with TB

*TB* Tuberculosis

#### Barriers to TB care-seeking

Participants were not aware and felt that they were not at risk for TB and therefore delaying care-seeking. The lack of awareness and the perceived risk was compounded by the absence of symptoms or features perceived to be present among people with TB, such as severe cough and appearing underweight. For some participants, the long-distance or inability to travel for services was a barrier to care-seeking.

*“I doubted I would have TB. I only had a cough for several days and was exhausted.” (IDI17, 64yo, male, urban, long delay)*

Participants with short delay reported the lack of perceived threat of TB to their health and competing priorities such as work and family commitments as barriers to TB care-seeking. Several participants with longer delay to diagnosis indicated care seeking at private health facilities or self-medication when symptoms surfaced, many of which occurred frequently and repetitively prior to TB diagnosis.

*“I didn’t go, I just didn’t care. Until it got really serious, then I go [to seek care]” (IDI11, 76yo, female, rural, short delay)*

*“I wanted to go as well. If I have a lot of time, I just wanted to go to the hospital to get treatment to be cured of that disease, and no longer sick. But I really didn’t have time, to be honest.” (IDI3, 37yo, male, urban, short delay)*

*“I never went to hospital. When I was [had] cough, I was buying [bought] medicines from pharmacy.” (IDI26, 68yo, male, rural, long delay)*

#### Facilitators to TB care-seeking

Participants from both groups expressed that encouragement from family members and other TB survivors was an impetus to care-seeking, especially when participants were symptomatic and ill. Participants also reported that success stories from TB survivors encouraged them to seek care.

“My mother saw me coughing, and she said that I should go for [TB] screening.” (IDI12, 33yo, male, urban, long delay)

Participants with shorter delays to diagnosis consistently reported that fear of infecting others and the ill effects of the conditions on wellbeing, work, and livelihoods prompted TB care-seeking. Many were worried that as they grew weaker, they were unable to go to work and the financial impact that would ensue. Furthermore, the economic woes worsened when significant out-of-pocket payment was required for medical consultations and medications in the private sector, therefore impelled care-seeking in public health facilities where TB services are available for free. Participants who experienced longer delay to diagnosis explained that they only sought care when TB symptoms became intolerable.

*“We don’t have enough money because when I got sick, I spent 2 thousand to 3 thousand dollars [on multiple consultations and treatments]. I couldn’t go to work. I stayed at home.” (IDI8, 32yo, female, rural, short delay)*

#### TB stigma

Another theme that emerged from both groups was perceived stigma against TB. Those with shorter delays to diagnosis indicated that they felt ashamed and embarrassed because of TB.

*“I was ashamed. I felt embarrassed because I had TB.” (IDI1, 50yo, male, urban, short delay)*

Many participants with longer delay to diagnosis reported both self-perceived and experience of discrimination perpetrated by people around them. Several accounts of participants distanced by members of their community after they were diagnosed were reported.

*“I always think like that. I thought that this disease is similar to HIV. People in my village discriminate against these diseases.” (IDI23, 58yo, male, rural, long delay)*

*“As I was sick, that one knows that I was sick, they didn’t dare to talk to us. They walked out while we walked in; so, it’s the effect, they know we like this [living with TB], they discriminated us.” (IDI12, 33yo, male, urban, long delay)*

## Discussion

In this study, we found that the median time from the onset of symptoms to TB diagnosis among people with TB in Cambodia was 49 days, and it is comparable to recent studies conducted in other high TB-burden countries in Asia [27, 28]. We found respondents living in rural areas of the country were more likely to experience a delay in diagnosis. This is consistent with findings from other low- and lower-middle-income countries [29–31], and high TB-burden settings [32]. Despite the similar risk profiles for TB between urban and rural areas in Cambodia [33], delayed TB diagnosis in rural Cambodia where large portions of the population are located [34] raised concerns regarding the differences in structural and social determinants between the dwellings in obtaining timely diagnosis and treatment. IDI participants from rural areas highlighted physical access and logistical barriers in TB care-seeking. However, specific challenges faced by the population living in rural areas should be further investigated. In Cambodia, different active case finding strategies targeting a specific setting and/or population have shown promising results in increasing case yields [4, 35], but the impact of these strategies on early identification of TB should be thoroughly examined. Our analyses also showed that participants who had education above the primary level and those with good TB knowledge (statistically not significant) had a shorter time to diagnosis, and it is in line with a recent meta-analysis on TB delayed diagnosis conducted among lower-middle-income countries [8]. We found significant correlations between TB knowledge and participants’ perception of the seriousness of TB (*P* <  0.001) and their self-perceived risk of contracting TB (*P* = 0.01) (Supplementary Table 1). This relationship was further explained by IDI participants as barriers to TB care-seeking. Participants who had lower education levels might have limited access to information about TB and therefore be less aware of the consequences of TB to themselves and their close contacts.

Our results showed that respondents who reported experiencing onset symptoms of hemoptysis and cough were less likely to delay their diagnosis. In conformity with two systematic reviews [8, 32], hemoptysis was perceived as more serious than cough, and people with these symptoms were more likely to seek care immediately. IDI participants who experienced longer delay to diagnosis also identified that care-seeking at public health facilities was only impelled when symptoms became unbearable. We found contrasting results for the onset symptoms of cough. Most studies reported a positive relationship between cough and delayed diagnosis as it overlaps with the manifestations of other respiratory infections and smoking [36–38]. Unfortunately, we did not collect data regarding concurrent respiratory infections during the onset of cough. Also, we did not find a significant correlation between smoking and cough in this study (*P* = 0.64).

Comparable to the finding from several systematic reviews [8, 32, 39], we found respondents who sought private health care and/or had self-medicated before TB diagnosis were 23% more likely to experience a longer time to diagnosis. The overarching theme of private health care seeking and/or self-medicating before TB diagnosis derived from IDIs was a prominent barrier to early diagnosis. In Cambodia, free TB services are largely provided by the public healthcare sector, where persons diagnosed with TB are registered and receive treatment. The private health providers mostly do not provide direct TB care and anti-TB medications, and the notification rate remains poor [15, 40]. However, 67% of the Cambodian population prefer to seek first treatment in the private healthcare sector due to ease of access, drugs supply, and responsiveness [41, 42], with more than two-thirds of the out-of-pocket payments for health care going to the private sector [43]. Hence, the private sector plays a significant role in the cascade of care, and revitalizing and contextualizing the public-private mix programs will be instrumental in finding the missing cases in Cambodia through referrals and public education [40, 44].

This study found that TB stigma contributed to delayed TB diagnosis and IDI participants further elaborated on the shame, embarrassment, and discrimination that they both perceived and experienced in the course of their TB care pathway. However, investigations on the relationship between stigma and diagnostic delay have produced mixed results [14]. In Thailand, the impact of TB stigma on time to diagnosis was found to be non-significant [45]. However, the effect of stigma on health-seeking behaviors was more pronounced in China [46], in accord with a 2014 systematic review [47]. The disagreements could be contributed by the use of different means of measurement [14]. As stigmatization is a cultural and social phenomenon [47], utilization of validated measurement tools [23] and incorporation of qualitative assessment of TB stigma will provide a more holistic insight into the role of stigma in affecting care-seeking behaviors. Nevertheless, the development of contextualized interventions aimed at addressing stigma will be pivotal in shortening the time to TB diagnosis.

The median time from TB diagnosis to treatment initiation was two days, and this finding showed that people with TB in Cambodia were timely treated in accordance with the national guideline (not exceeding three days from the day of diagnosis) [15]. In this study, 75% of the participants-initiated TB treatment within 3 days from the day of diagnosis. We did not find significant differences between urban and rural dwellers regarding treatment delay. This reflects the success of a decentralized DOTS program to all health centers in Cambodia, resulting in greater access and shorter delay to TB treatment [15, 48]. We found participants diagnosed with TB by smear microscopy were 50% more likely to experience a longer time to treatment initiation. This is congruent with a longer reporting time required by smear microscopy compared with GeneXpert MTB/RIF [49]. The utilization of point-of-care diagnostic tools such as GeneXpert MTB/RIF should be further optimized to improve case detection, timely diagnosis, and treatment of TB, and cost-effectiveness [50–52].

This study has several limitations. The onset of symptoms was self-reported by study participants and subjected to recall bias. To reduce the recall bias, we trained data collectors to interview participants within 1 month of diagnosis. Significant cultural and public holidays in Cambodia were used as prompts to aid recall and facilitate the estimation of the date of the onset of symptoms. Second, our sampling frame did not include all the ODs in Cambodia, hence limiting the generalizability of the findings to all people with TB in Cambodia. To assess the representativeness of our sample, we compared our findings with other nationally representative studies. Despite an overrepresentation of participants living in urban settings (36.9% vs 21.4% in the national census [34]), the ratio of men to women (1.3), and the proportion of bacteriologically confirmed TB by age groups (Supplementary Table 2) were comparable to the previous national TB prevalence survey [53]. The distance from home to the nearest public health facility reported in this study is also comparable to other studies conducted in Cambodia [33, 54, 55]. Hence, we believe the limitation on generalizability is minimal. In the assessment of private health care engagement and self-medication prior to TB diagnosis, we were not able to distinguish the frequency of these actions in this study. Therefore, we took the binary approach of either sought private health care and/or had self-medicated before TB diagnosis or not in the final analysis. To our knowledge, this is the first study to assess the extent and determinants of TB diagnostic and treatment delay in Cambodia. As there is no universal cut-off for diagnostic and treatment delay, we analyzed the outcome of interest in its continuous form to preserve as much information possible [56]. In the qualitative study, we undertook a long versus short delay analysis approach—with equal representation by gender and urban/rural dwellings for multifarious perspectives. We adopted the explanatory sequential design in this study for a comprehensive understanding of the findings, and the cogency of findings is further strengthened through the corroboration of both quantitative and qualitative data.

## Conclusions

In conclusion, we found that people with TB experienced substantial delays before being diagnosed with TB. Seeking private healthcare and self-medication before TB diagnosis; absence of TB symptoms such as cough, hemoptysis, and night sweats; stigma; rural residence; and a lower education level were responsible for longer delays. Delays in TB care-seeking for more than 2 months have shown to elevate the risk of household infection, and infectivity declined rapidly after the initiation of treatment [57]. Longer treatment delay was noted among TB diagnoses made using sputum-smear microscopy. Increasing public awareness about TB and consciousness regarding stigma, engaging the private healthcare providers, and tailoring approaches to target the rural areas could further improve early detection of TB and narrowing the gap of missing cases in Cambodia.

## Supplementary information

**Additional file 1.**

## Data Availability

The datasets used and/or analyzed during the current study are available from the corresponding author on reasonable request.

## Abbreviations

ACF: Active case finding
a*HR*: Adjusted hazard ratios
CENAT: National Center for Tuberculosis and Leprosy Control
*CI*: Confidence interval
DOTS: Directly observed treatment, short-course
GHQ: General health questionnaire
HIV: Human immunodeficiency virus
IDI: In-depth interview
IQR: Interquartile range
MTB/RIF: Mycobacterium tuberculosis/rifampicin resistance
NECHR: National Ethic Committee for Health Research
NUS IRB: National University of Singapore Institutional Review Board
OD: Operational district
PCF: Passive case finding
*SD*: Standard deviation
TB: Tuberculosis
USD: United States dollar
WHO: World Health Organization
