# Supplementary material for: Determinants of delayed diagnosis and treatment of tuberculosis in Cambodia: a mixed-methods study
Source: Infect Dis Poverty. 2020 May 7;9:49. doi: 10.1186/s40249-020-00665-8 (PMC7203857; doi:10.1186/s40249-020-00665-8)
Supplement: Supplementary file 1 — Additional file 1. [file 40249_2020_665_MOESM1_ESM.docx]

**Determinants of delayed diagnosis and treatment of tuberculosis in Cambodia: a mixed-methods study**

**Supplementary material**

**Contents**

[Measurement of TB knowledge 2](#_Toc38114300)

[Measurement of psychological distress 2](#_Toc38114301)

[In-depth interview guide 3](#_Toc38114302)

[Supplementary Table 1: Relationship between TB knowledge and the perception of TB 4](#_Toc38114303)

[Supplementary Table 2: Proportion of bacteriologically confirmed TB by age groups 4](#_Toc38114304)

[Supplementary Figure 1: Distribution of participants' age in years. 5](#_Toc38114305)

[Supplementary Figure 2: Persons with TB by age and sex. 5](#_Toc38114306)

[References 6](#_Toc38114307)

# **Measurement of TB knowledge**

We adapted the World Health Organization TB knowledge, attitude, and practices survey [1] to evaluate participants’ TB knowledge level based on 8 questions regarding characteristics, symptoms of TB, route of transmission, prevention, and treatment of TB. Each correct answer constituted one point with a total maximum score of 13. The median score of 9 was used as a cut-off, and participants were regarded as having poor TB knowledge if they scored the median and below and good TB knowledge if they scored above the median.

# **Measurement of psychological distress**

We utilized the General Health Questionnaire (GHQ)-12 [2] to identify psychological distresses from the day that the study participants experienced TB symptoms until TB was diagnosed based on the total score of the 6 negative items. Each item was measured on a Likert scale (0 to 4). We re-coded responses of 0 and 1 as 0 and responses of 2 and 3 as 1 according to the 0-0-1-1 scoring method [3]. The median score of 3 was used as a cut-off. The Cronbach’s alpha for GHQ-12 in this study was 0.814.

# **In-depth interview guide**

1. Knowledge, and perception of TB as a disease
   1. How are you feeling after being informed of the diagnosis? How has it affected you and your family?
   2. What do you know about TB?
   3. How do you think TB is transmitted?
   4. How can a person contract TB?
   5. How/Where do you think you were infected?
   6. How serious do you think TB is?
   7. Do you think if TB is a problem in Cambodia?
2. Experiences with seeking diagnosis and treatment for TB, with clinical and/or traditional approaches
   1. Can you describe your experience when you were sick and at what point did you decide to seek help?
   2. Where did you go to seek health care and why?
   3. How was the experience? Did you feel better after the visit?
   4. Can you describe where did you have your first consultation on your symptoms and your experience?
3. Barriers and facilitators experienced in seeking diagnosis and treatment
   1. Why wasn’t visiting the health center your first option?
      1. Prompts: Personal factors (knowledge, socio-economic, literacy), interpersonal factors (support systems in the family, stigma), community (perception of TB), organizational (distance to the health center, processes at the health center), social/policy (cultural norms, support for TB patients)
   2. At what point did you decide to visit the health center and why?
   3. If you have consulted another person (at the pharmacy, clinic, traditional healer, family/friends), what was their recommendation?

# **Supplementary Table 1: Relationship between TB knowledge and the perception of TB**

| **Variables** | **Poor TB knowledge^a^** | | **Good TB knowledge^a^** | | ***p*-value** |
| --- | --- | --- | --- | --- | --- |
|  | **Frequency** | **%** | **Frequency** | **%** |  |
| Perception about the seriousness of TB as a disease^b^ |  |  |  |  | <0.001 |
| Very serious | 43 | 24.3 | 232 | 42.8 |  |
| Not very serious | 134 | 75.7 | 310 | 57.2 |  |
| Self-perceived risk of getting TB^b^ |  |  |  |  | 0.011 |
| Yes, at-risk | 84 | 48.6 | 298 | 59.6 |  |
| No, not at-risk | 89 | 51.4 | 202 | 40.4 |  |

TB; tuberculosis

^a^Evaluated based on the answers from 8 questions regarding the characteristics, symptoms of TB, route of transmission, prevention, and treatment of TB with a total score of 13 (median = 9). Respondents were regarded as having poor TB knowledge if they scored the median and below and good TB knowledge if they scored above the median.

^b^Exclude missing values

# **Supplementary Table 2: Proportion of bacteriologically confirmed TB by age groups**

| **Age groups** | **Frequency** | **%** |
| --- | --- | --- |
| 15 - 24 | 6 | 2.1 |
| 25 - 34 | 14 | 4.9 |
| 35 - 44 | 32 | 11.3 |
| 45 - 54 | 52 | 18.3 |
| 55 - 64 | 85 | 29.6 |
| ≥ 65 | 96 | 33.8 |
| Total | 284 | 100 |


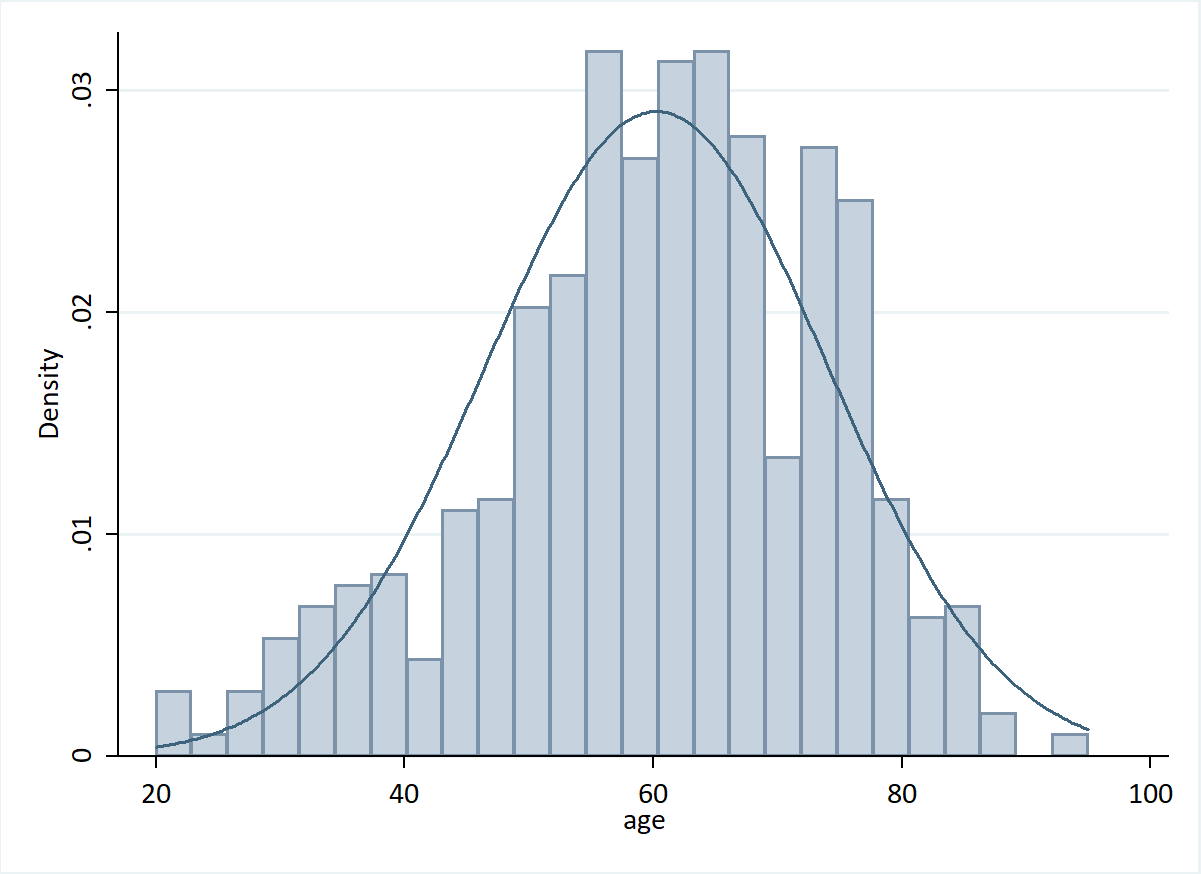


**Supplementary Figure 1: Distribution of participants' age in years.** The distribution of age in this study was left skewed (skewness *p*<0.001, kurtosis *p*=0.949, joint *p*<0.001).


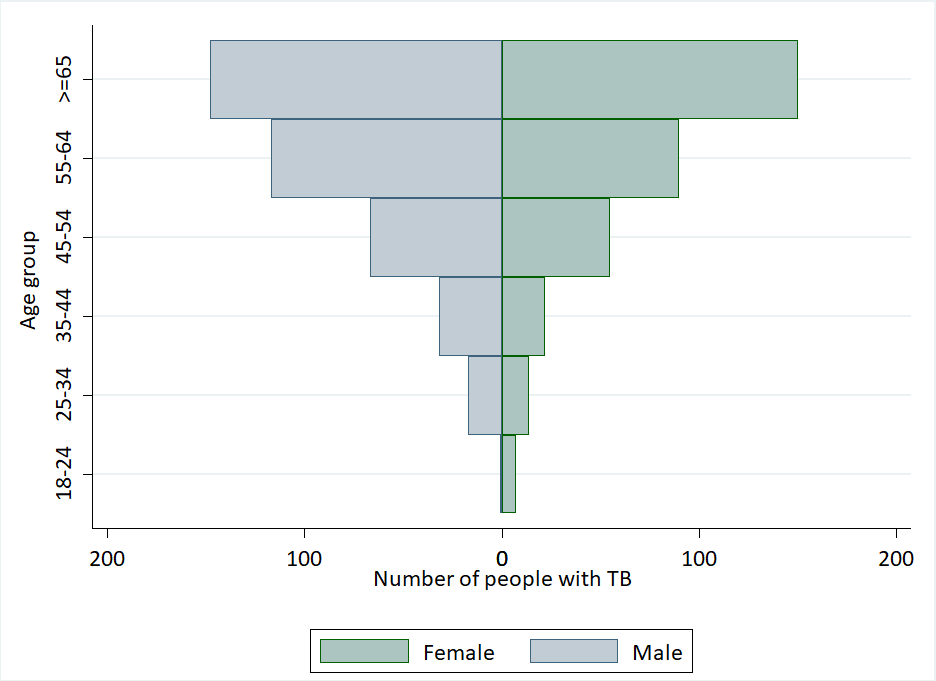


**Supplementary Figure 2: Persons with TB by age and sex.** Age and sex pyramid of study participants recruited in this study.

Most TB cases notified to the National TB Program comprised of persons aged above 55 and children. [4] We did not include children in our study. Otherwise, the age and sex distribution of our sample reflects the trends of cases notified to the National TB Program in 2018. [4]

# **References**

1. World Health Organization. Advocacy, communication and social mobilization for TB control: a guide to developing knowledge, attitude, and practice surveys. Geneva: World Health Organization; 2008.

2. Goldberg DP, Gater R, Sartorius N, Ustun TB, Piccinelli M, Gureje O, et al. The validity of two versions of the GHQ in the WHO study of mental illness in general health care. Psychol Med. 1997;27:191–7.

3. Goldberg D, Williams P. A user’s guide to the general health questionnaire. NFER-NELSON; 1988.

4. World Health Organization. TB country profile: Cambodia. Geneva: World Health Organization; 2019.
